# CCDC43 as a potential therapeutic target of Tian Yang Wan for the treatment of hepatocellular carcinoma by activating the hippo pathway

**DOI:** 10.3389/fonc.2023.1232190

**Published:** 2023-08-08

**Authors:** Mingyuan Tao, Dongwei Han, Siyu Wei, Changyu Gao

**Affiliations:** ^1^ Department of Prescription Science, Heilongjiang University of Chinese Medicine, Harbin, Heilongjiang, China; ^2^ College of Bioinformatics Science and Technology, Harbin Medical University, Harbin, China

**Keywords:** hepatocellular carcinoma, CCDC43, tumor microenvironment, Tian Yang Wan, hippo

## Abstract

**Introduction:**

Hepatocellular carcinoma (HCC) prevalence is rising annually, but the existing treatment strategies are limited; therefore, it is crucial to explore new therapeutic approaches.

**Methods:**

Here, we investigate the potential anti-cancer mechanism of an herbal medicine called Tian Yang Wan (TYW) in the treatment of HCC. The relationship of CCDC43 with immunity and cell death was analyzed by bioinformatics. Confirming the tumor suppressor effect of TYW on HCC cells by proliferation, invasion, migration and apoptosis assays

**Results:**

First, we analyzed by proteomics that CCDC43 expression was downregulated after TYW administration and promoted the hippo pathway. Then, a large sample's transcriptome study demonstrated that elevated CCDC43 expression was strongly correlated with clinical traits and a bad prognosis in HCC patients. Next, we observed through multiple advanced algorithms that CCDC43 is involved in a variety of oncology and immunology related pathways. Notably, we found higher tumor immune microenvironment with high CCDC43 expression. Furthermore, we demonstrated that CCDC43 is associated with immune checkpoints and found that it is a sensitive indicator of a large number of chemotherapeutic agents. Subsequently, we conducted experimental investigations to demonstrate the capacity of TYW to impede proliferation and migration, while inducing apoptosis in human HCC cell lines. Finally, we performed analysis of two cell death patterns which showed CCDC43 to be strongly correlated with multiple ferroptosis factors and cuproptosis factors.

**Discusion:**

In conclusion, our study comprehensively examined the prognostic, immunological, and therapeutic implications of CCDC43 in HCC, thereby elucidating the therapeutic mechanism of action in TYW.

## 1 Introduction

Hepatocellular carcinoma (HCC) is a prevalent gastrointestinal neoplasm. The conventional surgical interventions for HCC are categorized into therapeutic modalities and non-therapeutic modalities. Although transplantation is the most definitive treatment option, its feasibility is limited by the scarcity of organs (1). Traditional Chinese medicine (TCM) therapy emphasizes human-centeredness, which is an important part of clinical medicine and a characteristic of prevention and treatment of malignant tumors. Some studies have demonstrated that adjuvant therapy with TCM has been reported to increase the survival time among patients (2). Thus, how to find safe and effective drugs for the treatment of HCC, intervene and treat it at the early stage of tumor development, thus reducing the morbidity and mortality of HCC and prolonging the survival time of patients, has become an urgent problem to be solved.

Tian Yang Wan is the clinical experience formula of Professor Li Ji, the leader of the national key discipline of prescription medicine. Based on the theory of Chinese medicine, Professor Li Ji form the prescription “Tian Yang Wan”, which consists of Rehmannia, Morinda officinalis, Cistanche, Cynomorium songaricum, Cornus and Yam. It has the effect of warming the kidney yang and nourishing the kidney to benefit the essence. Under the guidance of clinical practice and TCM theory, Professor Li et al. applied TYW to the treatment of liver and kidney diseases and achieved good results. However, the potential anti-cancer mechanism of TYW has not been elucidated.

The Coiled-Coil Domain Containing (CCDC) family member, CCDC43, was studied to uncover its role in the control of invasion and metastasis of a variety of malignant cells (3). CCDC43 was found to act as a key player in several studies targeting gastric cancer. The expression of CCDC43 is a crucial factor in the growth and evolution of GC, which can lead to GC cell proliferation, invasion, and metastasis, thus resulting in a poor prognosis for patients (4). Upstream regulator HMGA1 promotes GC growth and metastasis by activating CCDC43 expression (5). Furthermore, high expression of CCDC43 is detected in more advanced oral squamous cell carcinoma (OSCC) and results in reduced anti-tumor immunity, leading to an increased metastatic capacity of OSCC cells (6). These findings indicate that CCDC43 may play a crucial role in regulating the immune infiltration profile and serve as a promising prognostic marker. However, the precise pathological function of CCDC43 remains unknown in HCC (7).

Our finding performed several bioinformatic and experimental analyses to investigate the mechanism of action of TYW in the treatment of HCC. The aim of this study was to fully characterize the expression pattern of CCDC43 in HCC. This was achieved by integrating proteomics, transcriptional profiling and clinical information. We analyzed prognostic features, tumor immune microenvironment characteristics, functional annotation and drug sensitivity prediction to explain the correlation between CCDC43 and HCC. Finally, it was verified that CCDC43 is closely associated with cell death patterns. Overall, CCDC43 has been identified as a promising drug candidate in TYW for the therapy of HCC by activating the hippo pathway, providing a precise therapeutic drug to combat HCC.

## 2 Materials and methods

### 2.1 Cell culture

Human HepG2 cells were purchased from Shanghai Fu Heng Biotechnology Co Ltd (Shanghai, China). The cell lines were cultured in DMEM (HyClone; GE Healthcare Life Sciences, Logan, UT, USA) supplemented with 1% penicillin and 1% streptomycin and 10% fetal bovine serum (FBS; HyClone; GEHealthcare Life Science), and the cells were maintained at 5% CO2 atmosphere maintained at 37°C.

### 2.2 Preparation of lyophilized powder of Tian Yang Wan

Tian Yang Wan combined Rehmannia, Morinda officinalis, Cistanche, Cynomorium songaricum, Cornus and Chinese yam in proportion. 1140 mL of purified water was added to the herbs, steeped, heated, and decocted continuously for 2 h, and the liquid was filtered out. Add 780mL of purified water to the container, decoct for 1.5 h, filter, and mix the two filtrates. The resulting aqueous decoction was filtered twice through 0.8 µmol filter membrane and lyophilized using a lyophilizer adjusted to automatic mode. The resulting lyophilized powder was sealed and stored in a -20°C refrigerator and autoclaved before use.

### 2.3 Proteomics

This study aimed to analyze the quantitative proteome of the samples using a combination of advanced technologies. The process involved protein extraction, enzymatic digestion, liquid chromatography-mass spectrometry analysis, and bioinformatics analysis. These techniques were integrated organically to efficiently and accurately determine the quantitative proteome of the samples.

### 2.4 Immune infiltration

The study utilized various methods to determine the abundance of immune and stromal cells, as well as the purity of tumor tissues. Three scores were obtained using the Estromal and Immune cells in Malignant Tumor tissues using Expression (ESTIMATE) algorithm (8). The extent of infiltration of six immune cell types in HCC were comprehensively analyzed using the Tumor Immune Estimation Resource 2.0 (TIMER2.0) web server (9). The Microenvironment Cell Populations-counter (MCPcounter) algorithm enabled the identification of ten distinct types of immune cells present in the tumor microenvironment (TME) (10). Furthermore, the levels of 28 immune cell types were available with single sample genomic enrichment analysis (ssGSEA). To assess the immune status of HCC, 74 immune modulators were accessed from two publications (11, 12). Analysis of CCDC43 response to anti-PD1 and anti-CTLA4 therapies by subclass mapping (SubMap) (13).

### 2.5 Functional annotation

The Kyoto Gene and Genome Encyclopedia (KEGG) genes were downloaded from the MSigDB database (14). To analyze the functional pathways, the R packages clusterProfiler and GSVA were used (15, 16).

### 2.6 Drug response estimation

To predict the drug sensitivity of patients, pharmacogenomic data were obtained from the Genomics of Drug Sensitivity in Cancer (GDSC) database (https://www.cancerrxgene.org/). The R package pRRophetic was used to calculate the half-maximal inhibitory concentrations (IC50) of the drugs.

### 2.7 Statistical analysis

Patients were divided into high and low groups based on the abundance of CCDC43 expression. Kaplan-Meier curves (KM curves) were generated to estimate survival. KM curves were implemented using the R package survminer. Uni-Cox and multi-Cox regression analyses were performed to assess hazard ratios. The R package survival was utilized for cox regression analysis. Correlation coefficients were calculated using Pearson correlation analysis. Visualization of most data was done with the R package ggplot2. Somatic mutation analysis results were generated by the R package maftools (17). Tumor mutation burden (TMB) is used to calculate the TMB value of the dataset with the R package maftools. Heat maps were generated with the R package complexHeatmap (18). All statistics and plots were performed using R version 4.1.2 (https://www.r-project.org). *P < 0.05, **P < 0.01, ***P < 0.001, ****P < 0.0001.

### 2.8 CCK8 assay to determine cell proliferation

HepG2 cells in logarithmic growth phase were added to FBS DMEM and cell suspension. At various time points, CCK-8 solution (biosharp) was added according to the instructions. After incubating the cells for another 2 hours in an incubator protected from light, the absorbance at 450 nm was read using an enzyme marker, and the curves were plotted.

### 2.9 Transwell assay

Cell Migration. 12% FBS was added to a 24-well plate combined with Transwell and then incubated for 0.5 h. HepG2 cells were injected on a 6-well plate, digested, processed by centrifugation, and then new culture solution was added again, stirred, and finally a small amount of cell suspension was taken and repeated 3 times to calculate the mean value. After 24 hours, the solution was removed, and the chambers were washed three times using PBS. Finally, HepG2 cells were thoroughly cleaned with a cotton swab. The samples were immersed in fixative solution for 10 min, removed immediately, poured into staining solution, immersed again for 15 min, and rinsed five times with PBS buffer. After complete evaporation of the bottom membrane, counting and photographing were performed for documentation.

Cell Invasion. Thaw the stock solution 1h in advance, then transfer it to an ice box and place the 1.5 mL EP tube, gun tip and cell chambers in the freezer. Remove 40 µL/well of ECM Gel usage solution from the ice and slowly pour it into the top of the celllet. The cells were placed in a 37°C incubator, digested, centrifuged and counted, and then diluted to obtain a cell suspension. Subsequently, 200µL of the suspension was mixed into the medium and transplanted into a 37°C dish with a final addition of 10% FBS+ medium. 2 h later, the liquid was removed from the upper chamber and cleaned twice using PBS. Crystalline violet dye was poured into the upper layer for staining for 5 min. Using a microscope, the cell chambers were inverted and photographed with a 100x field of view, and the thickness of the membrane was measured and the mean value calculated.

### 2.10 Annexin v-FITC/PI flow cytometry

Cells were cleaned using PBS technique and 5*Binding Buffer was diluted to 1*Binding Buffer, then 500 µL 1*Binding Buffer was mixed with 5 µL annexinv-FITC and 10 µL PI dye, placed into a suitable environment and allowed to incubate for 5 in in a shaded environment so that the cells could be finely analyzed using flow cytometry.

## 3 Results

### 3.1 TYW represses the oncogenic CCDC43 and activates the hippo pathway

**Figure 1:**
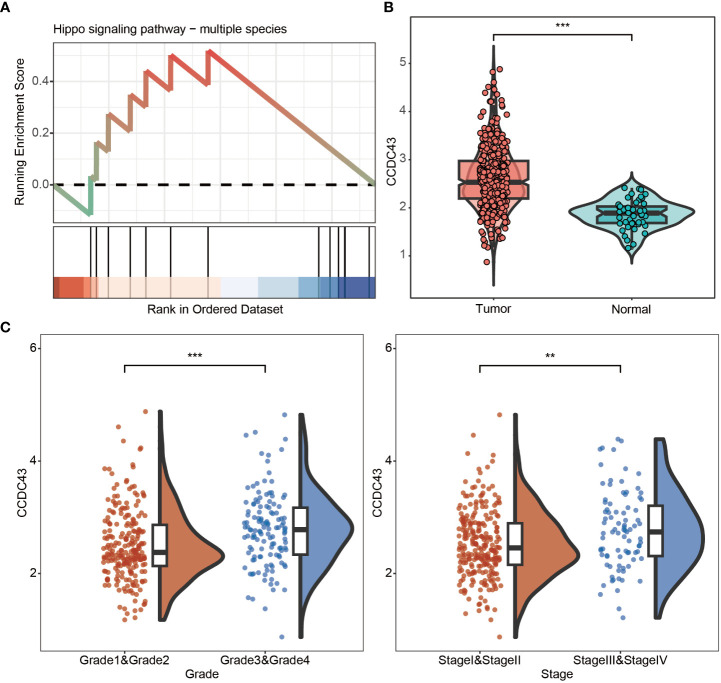
Proteomic results and expression characteristics of CCDC43. **(A)** GSEA map of hippo pathway after TYW administration **(B)** CCDC43 expression levels in HCC and normal samples. **(C)** Differential expression of CCDC43 in different grades and stages in the transcriptome dataset. **P < 0.01, ***P < 0.001.

We first analyzed the proteomics and found that CCDC43 expression was reduced after TYW administration. Next GSEA analysis confirmed the function of TYW to promote the hippo pathway (**Figure 1A**). Next, based on large sample transcriptome data, we analyzed the transcript abundance of CCDC43 in HCC and identified a malignancy-related overexpression pattern of CCDC43 (**Figure 1B**). In addition, the transcript levels of CCDC43 were positively correlated with Grade and Stage, suggesting that CCDC43 expression was enhanced at later stages of tumor growth and at greater extent of dissemination (**Figure 1C**). These results indicate that CCDC43 expression levels increase as HCC progresses.

### 3.2 Overexpression of CCDC43 corresponds to bad survival in HCC patients

**Figure 2:**
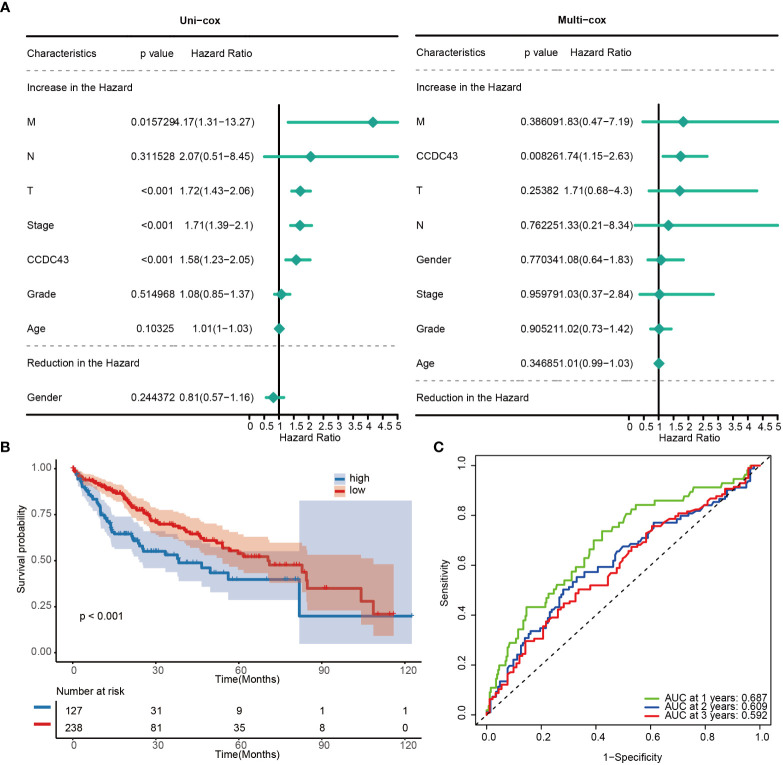
Prognostic potential of CCDC43. **(A)** A forest plot has been created to display both uni- and multi- cox proportional hazard ratios for CCDC43. **(B)** Kaplan-Meier curves for two groups. **(C)** Receiver operating characteristic curves for CCDC43.

Next, we looked into CCDC43’s predictive significance in HCC. The findings of the analysis suggested that CCDC43 was identified as a risk factor for death in HCC, as shown by single Cox and multiple Cox analyses (**Figure 2A**). The KM curve of transcriptome data based on Kaplan-Meier analysis subsequently made a compelling case for the decreased survival rate of HCC patients with high CCDC43 expression (**Figure 2B**). AUC values greater than 0.592 for 1, 2, and 3 years were seen in the time-dependent receiver operating characteristic (ROC) curve of CCDC43, which also demonstrated good sensitivity and specificity (**Figure 2C**). In conclusion, in patients with HCC, higher CCDC43 transcript abundance tends to correspond to a higher risk of death. Therefore, CCDC43 can be used as a strong clinical indicator of HCC.

### 3.3 CCDC43 is involved in HCC progression and immune regulation

**Figure 3:**
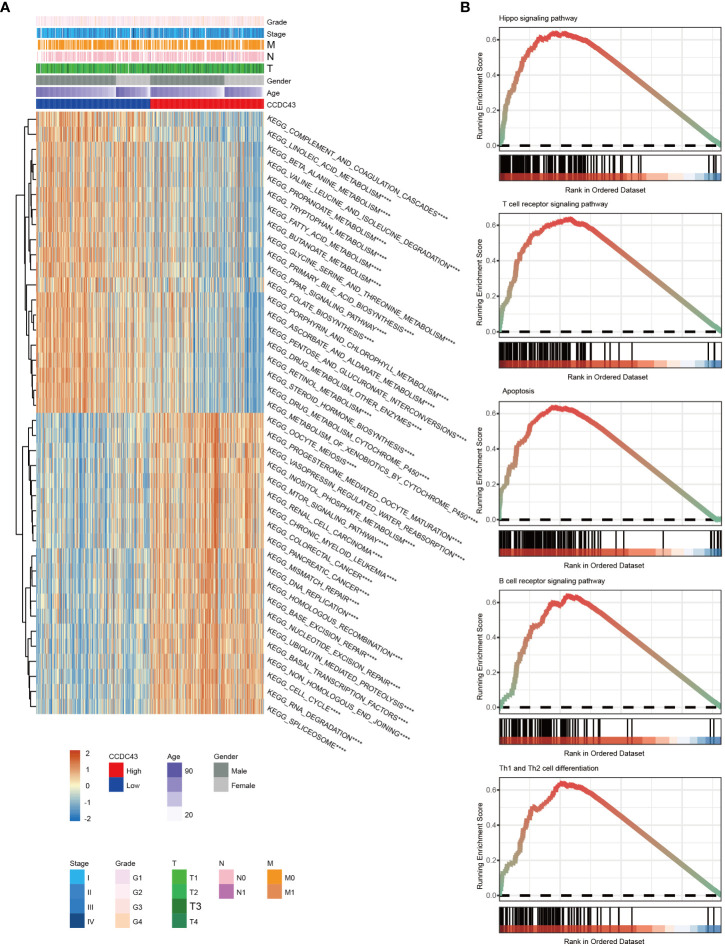
Functional annotation of CCDC43 expression. **(A)** A heat map has been generated to display the gene set variation analysis results of CCDC43. **(B)** GSEA plots have been generated displaying several signaling pathways that positively correlate with CCDC43 expression levels. ****P < 0.0001.

We used a GSVA analysis to investigate CCDC43’s role as an oncogene. The findings showed that CCDC43 is involved in multiple important pro-carcinogenic activities, including abnormal activity of the NOTCH signaling network, mTOR signaling route, inositol phosphate metabolism, ubiquitin-mediated proteolysis, and hippo signaling system (**Figure 3A**). The crucial involvement of CCDC43 in the tumor immune microenvironment, including T cell receptor signaling route, B cell receptor signaling pathway, apoptosis, and Th1 and Th2 cell differentiation, was further verified by GSEA analysis (**Figure 3B**). Overall, CCDC43 amplification provides a favorable ecological environment for HCC progression.

### 3.4 CCDC43 expression is associated with different genomic variants

**Figure 4:**
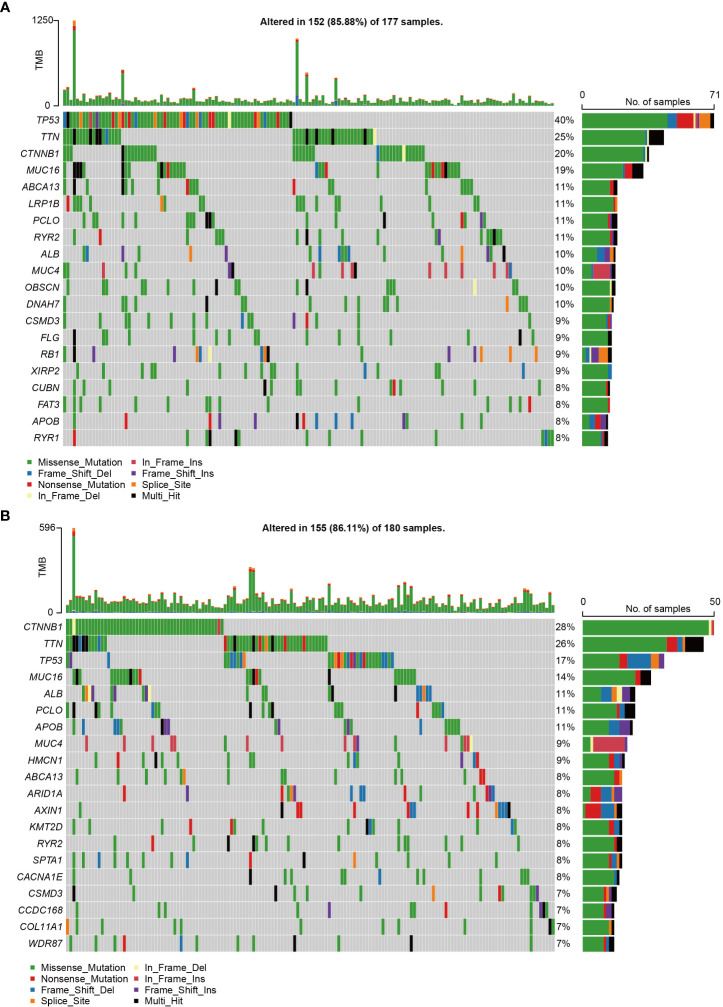
CCDC43-related genomic changes were found in HCC samples. **(A)** HCC with high expression of CCDC43 was shown to have somatic mutations. **(B)** HCC with reduced CCDC43 expression and somatic mutations.

We conducted somatic mutation analysis on the HCC dataset to investigate the putative genetic regulatory function of CCDC43. The global perspective of mutation distribution revealed that both the high and low CCDC43 groups had a high prevalence of mutations in the cellular tumor antigens p53 (TP53), titin (TTN), and catenin beta 1 (CTNNB1) (**Figures 4A, B**). Mucin 16 (MUC16) (19%), ATP binding cassette subfamily A member 13 (ABCA13) (11%), and lipoprotein receptor-related protein 1B (LRP1B) (11%) were the three most commonly altered genes in the high CCDC43 group (**Figure 4A**). In contrast, in the low CCDC43 group were albumin (ALB) (11%), piccolo presynaptic cytomatrix protein (PCLO) (11%) and apolipoprotein B (APOB) (11%) (**Figure 4B**). In addition, TMB levels were significantly higher in the CCDC43 high expression group (**Supplementary Figure S1**), and patients were also more likely to benefit from immunotherapy. In summary, these results present a CCDC43-based genomic landscape.

### 3.5 CCDC43 related to multiple immune infiltrating cells

**Figure 5:**
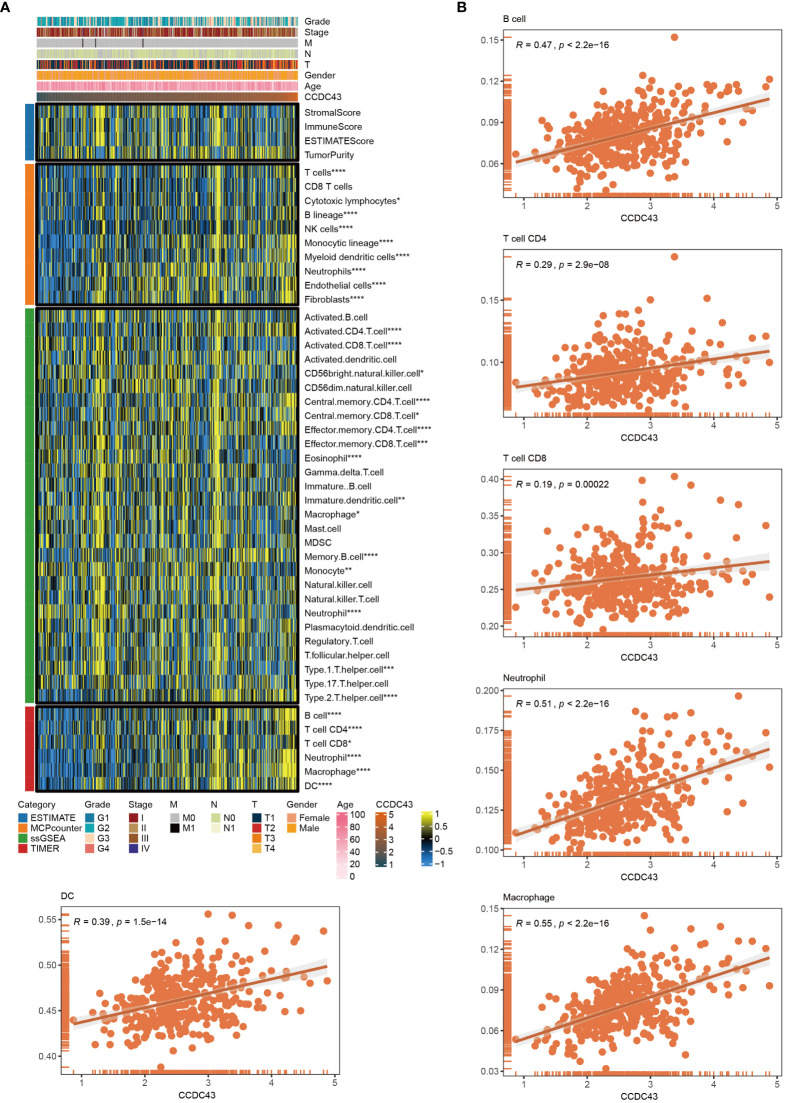
Role of CCDC43 in the immunity. **(A)** A heat map was generated to visualize the concatenation of CCDC43 expression levels with the abundance of the infiltrating immune cell abundance using ESTIMATE, MCPcounter, ssGSEA, and TIMER algorithms. **(B)** A corrplot was generated to show the relationship between CCDC43 gene expression and B cell, CD4 T cell, CD8 T cell, neutrophil, macrophage, and DC levels for TIMER data. *P < 0.05, **P < 0.01, ***P < 0.001, ****P < 0.0001.

Based on the transcriptomic dataset, immune cell abundance was determined using ESTIMATE, MCP counter, ssGSEA and TIMER, given the significant role they play in TME (**Figure 5A**). It is worth noting that the transcript abundance of CCDC43 is proportional to the abundance of various immune cells. Further, an extensive investigation of the correlation between CCDC43 and B cell, CD4 T cell, CD8 T cell, neutrophil, macrophage, and dendritic cell (DC) infiltration was performed using data from and TIMER (**Figure 5B**). Higher CCDC43 expression levels were correlated with anti-PD-1 immunotherapy response according to SubMap analysis (**Supplementary Figure S2**).

### 3.6 CCDC43 is a potential immunotherapeutic and drug target in HCC patients

**Figure 6:**
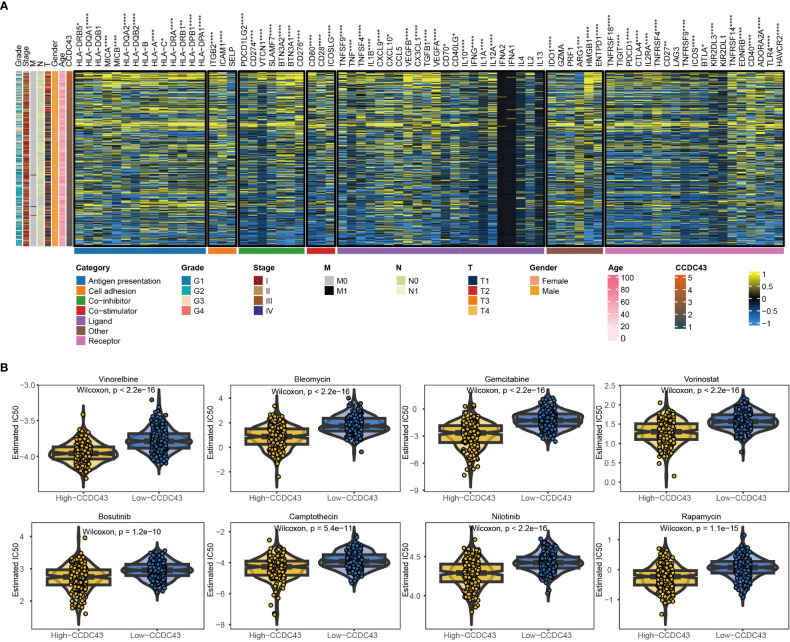
Potential immune checkpoint therapy and chemotherapy targets involved in CCDC43 for HCC. **(A)** Correlation of CCDC43 with seven immune processes in HCC. **(B)** Box plots were created to represent the predicted IC50 values of 10 drugs for HCC in two groups. *P < 0.05, **P < 0.01, ***P < 0.001, ****P < 0.0001.

We then conducted a correlation analysis between CCDC43 and various immune processes involved in antigen presentation, cell adhesion, co-inhibition or co-stimulation, and ligand-receptor interactions (19). **Figure 6A** showed a close association between CCDC43 and a majority of immune modulators in HCC, like PD-1, CTLA4, TIGIT, IFNG, and VEGFA. Together, these data suggest that targeting CCDC43 could exert a co-operative influence with existing pharmacological treatment modalities and promote anti-HCC sensitivity.

The GDSC database was used to evaluate drug response in two groups in order to investigate drug sensitivity. Our findings indicated that the high group of HCC patients showed a clearly decreased drug responses to AZD8055, Bleomycin, Bosutinib, Camptothecin, Gemcitabine, Methotrexate, Nilotinib, Rapamycin, Vinorelbine, and Vorinostat, as evidenced by the significantly lower IC50 values compared to the other group (**Figure 6B**). The effectiveness of the pharmacological approaches mentioned here warrants additional study.

### 3.7 TYW exhibits tumor suppressive effects in HCC cells

**Figure 7:**
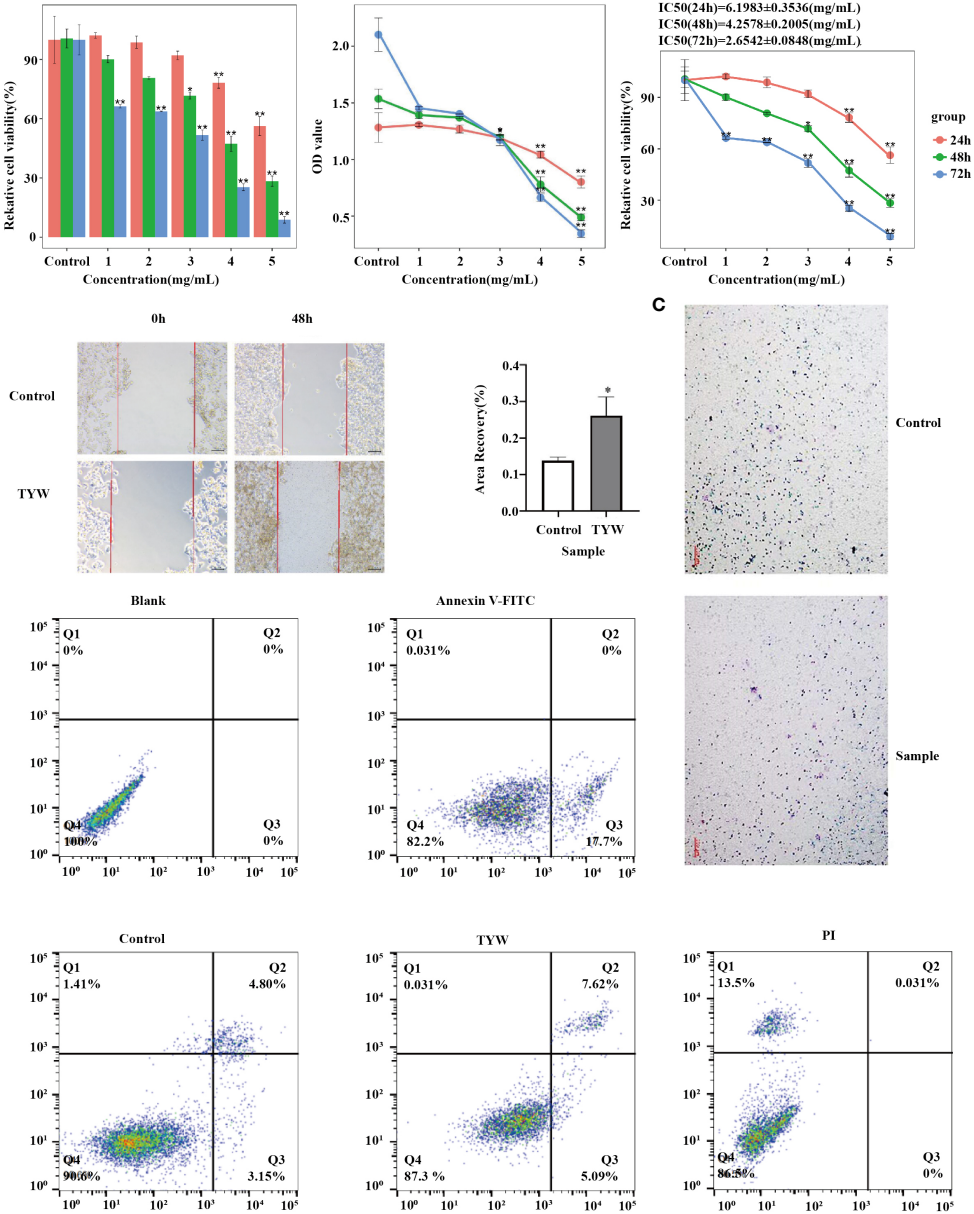
Proliferation, migration, invasion and apoptosis of HCC cells. **(A)** HCC cell line HepG2 was cultured with TYW, and cell proliferation and toxicity were detected by CCK-8 assay. **(B)** Transwell assay to detect the migration ability of HCC cell line HepG2. **(C)** Detection of the invasive ability of TYW on HepG2. **(D)** Flow cytometry to detect apoptosis of HepG2 cells. *P < 0.05, **P < 0.01.

Cancer cell growth rate was found to be significantly suppressed upon treatment with TYW, as observed from the results of CCK-8 and colony formation assays (**Figure 7A**; **Supplementary Table S1**). Furthermore, we also explored the impact of TYW on the migration and invasion of cancer cells. The number of cells migrating through the membrane to the bottom of the pore was markedly diminished and the metastasizing cells through the matrix gel layer when compared with the empty carriers (**Figures 7B, C**). The results showed that TYW promoted apoptosis (**Figure 7D**).

### 3.8 CCDC43 is significantly associated with two patterns of cell death

**Figure 8:**
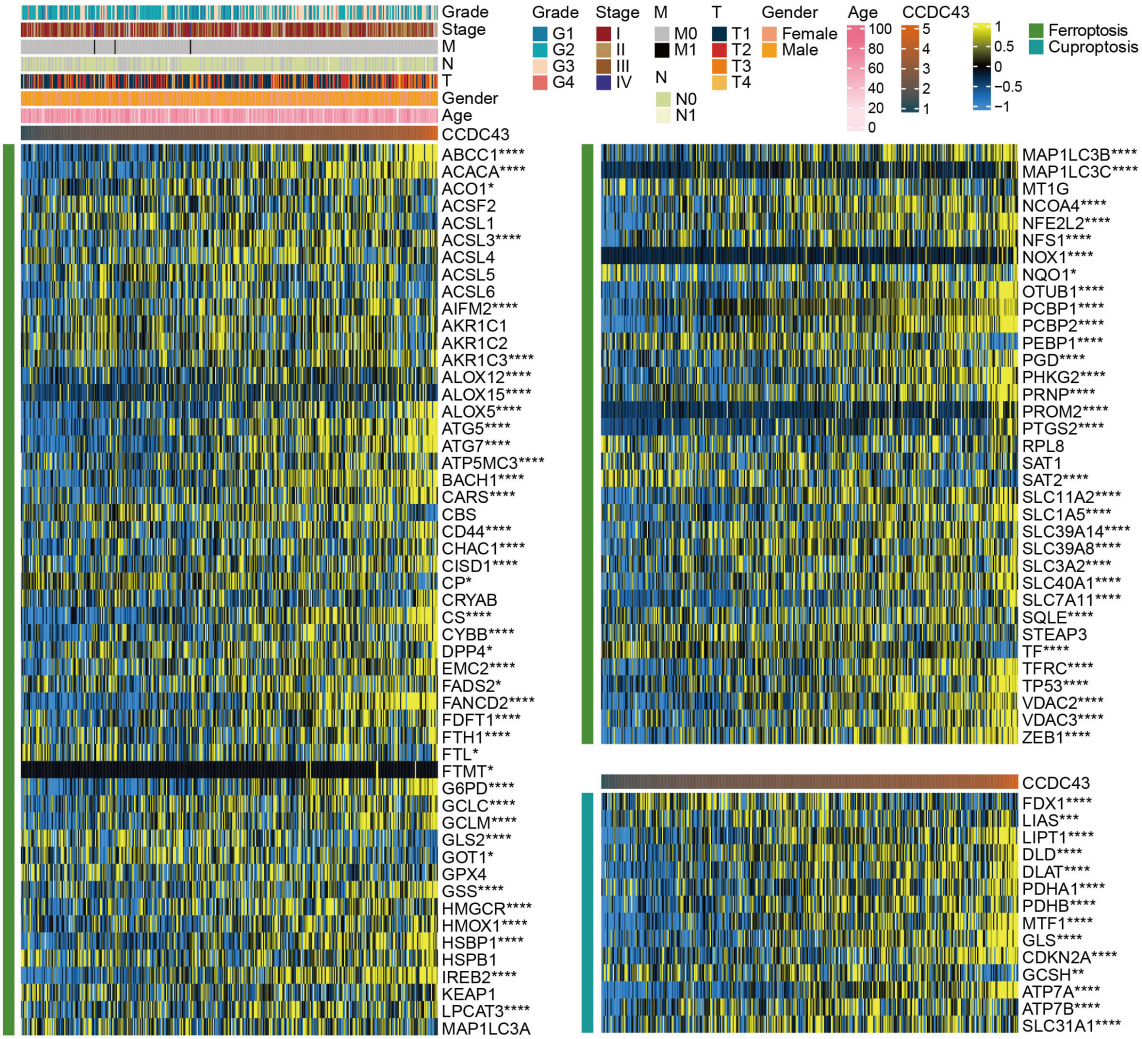
Heat map of the correlation of CCDC43 with 87 ferroptosis factors and 14 cuproptosis factors in HCC. *P < 0.05, **P < 0.01, ***P < 0.001, ****P < 0.0001.

Numerous studies have highlighted the critical importance of ferroptosis and cuproptosis in tumor suppression, which is of strategic importance for cancer treatment (20, 21). We retrieved 87 ferroptosis genes and 14 cuproptosis genes from a publication to analyze their correlation with CCDC43 expression (22). The results demonstrated that 82.8% (72/87) of the ferroptosis genes and all cuproptosis genes were significantly associated with CCDC43. Among them, positive correlation factors account for the majority, such as ABCC1, ATG5, TP53, and PDHA1 (**Figure 8**). Therefore, the higher the expression of CCDC43, the more it promotes cell death, and there is great potential to target it in cancer therapy.

## 4 Discussion

The incidence of HCC is rising steadily each year, but treatment options are limited (23). The development of novel drugs and the systematic application of newly discovered biomarkers will contribute to the treatment of HCC. The purpose of this study was to elucidate the pharmaceutical function of TYW against HCC by proteomic analysis combined with bioinformatics analysis, that is, the reduction of CCDC43 expression and promotion of hippo signaling pathway after drug administration. We found that CCDC43 transcript levels were positively related to the clinical features of HCC, while patient survival was negatively correlated. Additionally, our results showed that CCDC43 is linked to the tumor immune microenvironment of HCC and demonstrated that CCDC43 may also be a therapeutic target for checkpoint inhibitors. Moreover, TYW was experimentally confirmed to attenuate a variety of tumor characteristics, which provides a viable option for future treatment. By analyzing cell death patterns, we concluded that elevated expression of CCDC43 contributes to the promotion of cell death.

Coiled-coil domain-containing (CCDC) was discovered as early as 2001 (24). This family is present in intercellular transmembrane signaling (25), transcriptional regulation and AKT signaling pathway (26), among other functions, while CCDC has been shown to be associated with the pathogenesis of many cancers (3, 27, 28). Moreover, increased expression of CCDC43 is correlated with decreased anti-tumor immunity and heightened DNA damage repair capability, which could be a trigger for inducing epithelial-mesenchymal transition. Therefore, we believe that the functional mechanisms associated with CCDC43 in HCC deserve further exploration using experimental approaches and bioinformatics analysis. During our study, we discovered that CCDC43 is involved in regulating several pathways related to both tumor and immune processes, including the hippo pathway. According to previous research, CCDC43 is known to have a regulatory function upstream of mTORC1 (29), which was likewise confirmed in our study. Our findings suggest that CCDC43 may disrupt TME homeostasis not only through oncogenic pathways but also through multiple immune modalities (30). The transcript levels of CCDC43 were strongly and positively correlated with multiple cell death pattern-related genes, and we have reason to believe that CCDC43 is an important factor constituting cell death.

From the results of multiple immunological analyses, our findings revealed that upregulation of CCDC43 expression significantly augmented the frequency of multiple immune cells such as neutrophils and macrophages. These findings suggest a critical function for CCDC43 in modulating the immune microenvironment in HCC. Furthermore, we demonstrated that high expression of CCDC43 was concomitant with elevated expression of various immune checkpoint genes, for example, HLA-DQA1, HLA-DRA, ITGB2, ICAM1, CD80, CD28, TNFSF9, IL10, IFNG, PDCD1, and CTLA4. These results show the strategic importance of CCDC43 in immunotherapy. In addition to these, we found that CCDC43 was significantly associated with almost all of the collected cell death-related genes. As the expression level of CCDC43 increased, the expression of ferroptosis and cuproptosis related genes also showed the same trend. Although there have been no reports showing the role of CCDC43 in cell death in the context of HCC, it was previously shown in a gastric cancer study that CCDC43 acts opposite to four and a half LIM domains 1 (FHL1), which in turn promotes cell death (31). Researchers have targeted phosphoseryl-tRNA kinase (PSTK) to inhibit the ferroptosis process in HCC cells, thereby increasing drug sensitivity. Notably, Fengyuan Tang et al. found that YAP/TAZ could inhibit the ferroptosis process. Since the Hippo pathway also contains YAP/TAZ as a key link (32), it is reasonable to believe that TYW has similar effects and is expected to improve the therapeutic efficacy of HCC.

In the process of rapid growth, tumors often over-consume nutrients and energy in the body, so that tumor patients often have deficiency of qi and blood, and weakness of positive qi, etc. Therefore, Chinese medicine often applies the treatment method of strengthening spleen and benefiting qi to protect the positive qi of human body to resist the growth and invasion of tumors. According to Chinese medicine, “deficiency of vital energy” is the root cause of tumor disease. In the formula of TYW, Rehmannia and Morinda officinalis are the rulers. Cistanches are sweet and salty and warm in nature, and pharmacological studies have shown that they have anti-tumor effects (33). Cynomorium songaricum is sweet and warm in nature and has anti-viral, anti-obesity and anti-diabetic effects (33, 34). In addition, the sour and warm Cornus officinalis has the function of nourishing the liver and kidney, fixing the essence and reducing urine. One of the main active ingredients of Cornus officinalis is Cornus polysaccharide, whose effects include anti-tumor, anti-aging, and immune function enhancement, etc. (35). The Chinese yam is sweet and calm and can nourish the spleen and kidney and benefit yin.

Although we verified through some experiments that TYW inhibited the proliferation of tumor cells, the specific pharmacological mechanism is not clear. Besides, as this study primarily utilized open online databases for data analysis, further validation using clinical data would be necessary. Traditional Chinese medicine as a human world medicine, is the crystallization of the Chinese traditional culture wisdom, but because of the prescriptions of Chinese medicine, Chinese medicine pharmacology discipline own complexity. The study of Chinese medicine treatment of tumor has yet to dig deeper and continue to explore. It is hoped that this article can provide reference for more researchers in the field of TCM cancer prevention and treatment.

## Data availability statement

The original contributions presented in the study are included in the article/**Supplementary Material**. Further inquiries can be directed to the corresponding authors.

## Author contributions

MT and DH contributed equally as co-first authors. Conceptualization and methodology, MT and DH. Software, SW. Validation and data management, MT. Preparation of original draft, MT and DH. Supervision, SW and CG. Project management, DH. All authors have read and agreed to the published version of the manuscript.

## Supplementary material

Supplementary Figure 1 Box plot of TMB levels between high and low CCDC43 expression groups.

Supplementary Figure 2 SubMap analysis of CCDC43 expression levels in HCC.

Supplementary Table 1 Cell proliferation and toxicity assayed by CCK-8 assay.

## References

[1] Ganesan P Kulik LM. Hepatocellular carcinoma: New developments. Clin Liver Dis (2023) 27(1):85–102. doi: 10.1016/j.cld.2022.08.004 36400469

[2] Liu X Li M Wang X Dang Z Yu L Wang X. Effects of adjuvant traditional Chinese medicine therapy on long-term survival in patients with hepatocellular carcinoma. Phytomedicine (2019) 62:152930. doi: 10.1016/j.phymed.2019.152930 31128485

[3] Guo L Li B Lu Z Liang H Yang H Chen Y. CCDC137 is a prognostic biomarker and correlates with immunosuppressive tumor microenvironment based on pan-cancer analysis. Front Mol Biosci (2021) 8:674863. doi: 10.3389/fmolb.2021.674863 34055889 PMC8155610

[4] Wang J Wu X Dai W Li J Xiang L Tang W. The CCDC43-ADRM1 axis regulated by YY1, promotes proliferation and metastasis of gastric cancer. Cancer Lett (2020) 482:90–101. doi: 10.1016/j.canlet.2020.03.026 32278016

[5] Yang Q Wang Y Li M Wang Z Zhang J Dai W. HMGA1 promotes gastric cancer growth and metastasis by transactivating SUZ12 and CCDC43 expression. Aging (Albany NY) (2021) 13(12):16043–61. doi: 10.18632/aging.203130 PMC8266323 34167089

[6] Wang Z Zhang H Zhai Y Li F Shi X Ying M. Single-cell profiling reveals heterogeneity of primary and lymph node metastatic tumors and immune cell populations and discovers important prognostic significance of CCDC43 in oral squamous cell carcinoma. Front Immunol (2022) 13:843322. doi: 10.3389/fimmu.2022.843322 35401551 PMC8986980

[7] Wei S Tao J Xu J Chen X Wang Z Zhang N. Ten years of EWAS. Adv Sci (Weinh) (2021) 8(20):e2100727. doi: 10.1002/advs.202100727 34382344 PMC8529436

[8] Yoshihara K Shahmoradgoli M Martinez E Vegesna R Kim H Torres-Garcia W. Inferring tumour purity and stromal and immune cell admixture from expression data. Nat Commun (2013) 4:2612. doi: 10.1038/ncomms3612 24113773 PMC3826632

[9] Li T Fu J Zeng Z Cohen D Li J Chen Q. TIMER2.0 for analysis of tumor-infiltrating immune cells. Nucleic Acids Res (2020) 48(W1):W509–W14. doi: 10.1093/nar/gkaa407 PMC7319575 32442275

[10] Becht E Giraldo NA Lacroix L Buttard B Elarouci N Petitprez F. Erratum to: Estimating the population abundance of tissue-infiltrating immune and stromal cell populations using gene expression. Genome Biol (2016) 17(1):249. doi: 10.1186/s13059-016-1113-y 27908289 PMC5134277

[11] Schreiber RD Old LJ Smyth MJ. Cancer immunoediting: integrating immunity's roles in cancer suppression and promotion. Science (2011) 331(6024):1565–70. doi: 10.1126/science.1203486 21436444

[12] Zhang M Wang X Chen X Zhang Q Hong J. Novel immune-related gene signature for risk stratification and prognosis of survival in lower-grade glioma. Front Genet (2020) 11:363. doi: 10.3389/fgene.2020.00363 32351547 PMC7174786

[13] Hoshida Y Brunet JP Tamayo P Golub TR Mesirov JP. Subclass mapping: identifying common subtypes in independent disease data sets. PloS One (2007) 2(11):e1195. doi: 10.1371/journal.pone.0001195 18030330 PMC2065909

[14] Liberzon A Birger C Thorvaldsdottir H Ghandi M Mesirov JP Tamayo P. The Molecular Signatures Database (MSigDB) hallmark gene set collection. Cell Syst (2015) 1(6):417–25. doi: 10.1016/j.cels.2015.12.004 PMC4707969 26771021

[15] Yu G Wang LG Han Y He QY. clusterProfiler: an R package for comparing biological themes among gene clusters. OMICS. (2012) 16(5):284–7. doi: 10.1089/omi.2011.0118 PMC3339379 22455463

[16] Hanzelmann S Castelo R Guinney J. GSVA: gene set variation analysis for microarray and RNA-seq data. BMC Bioinf (2013) 14:7. doi: 10.1186/1471-2105-14-7 PMC3618321 23323831

[17] Mayakonda A Lin DC Assenov Y Plass C Koeffler HP. Maftools: efficient and comprehensive analysis of somatic variants in cancer. Genome Res (2018) 28(11):1747–56. doi: 10.1101/gr.239244.118 PMC6211645 30341162

[18] Gu Z Eils R Schlesner M. Complex heatmaps reveal patterns and correlations in multidimensional genomic data. Bioinformatics (2016) 32(18):2847–9. doi: 10.1093/bioinformatics/btw313 27207943

[19] Li S Zhang N Liu S Zhang H Liu J Qi Y. ITGA5 is a novel oncogenic biomarker and correlates with tumor immune microenvironment in gliomas. Front Oncol (2022) 12:844144. doi: 10.3389/fonc.2022.844144 35371978 PMC8971292

[20] Zhang C Liu X Jin S Chen Y Guo R. Ferroptosis in cancer therapy: a novel approach to reversing drug resistance. Mol Cancer (2022) 21(1):47. doi: 10.1186/s12943-022-01530-y 35151318 PMC8840702

[21] Tang D Chen X Kroemer G. Cuproptosis: a copper-triggered modality of mitochondrial cell death. Cell Res (2022) 32(5):417–8. doi: 10.1038/s41422-022-00653-7 PMC9061796 35354936

[22] Zou Y Xie J Zheng S Liu W Tang Y Tian W. Leveraging diverse cell-death patterns to predict the prognosis and drug sensitivity of triple-negative breast cancer patients after surgery. Int J Surg (2022) 107:106936. doi: 10.1016/j.ijsu.2022.106936 36341760

[23] Yang JD Hainaut P Gores GJ Amadou A Plymoth A Roberts LR. A global view of hepatocellular carcinoma: trends, risk, prevention and management. Nat Rev Gastroenterol Hepatol (2019) 16(10):589–604. doi: 10.1038/s41575-019-0186-y 31439937 PMC6813818

[24] Burkhard P Stetefeld J Strelkov SV. Coiled coils: a highly versatile protein folding motif. Trends Cell Biol (2001) 11(2):82–8. doi: 10.1016/S0962-8924(00)01898-5 11166216

[25] Liu Z Yan W Liu S Liu Z Xu P Fang W. Regulatory network and targeted interventions for CCDC family in tumor pathogenesis. Cancer Lett (2023) 216225. doi: 10.1016/j.canlet.2023.216225 37182638

[26] Li CF Wu WR Chan TC Wang YH Chen LR Wu WJ. Transmembrane and coiled-coil domain 1 impairs the AKT signaling pathway in urinary bladder urothelial carcinoma: A characterization of a tumor suppressor. Clin Cancer Res (2017) 23(24):7650–63. doi: 10.1158/1078-0432.CCR-17-0002 28972042

[27] Radulovich N Leung L Ibrahimov E Navab R Sakashita S Zhu CQ. Coiled-coil domain containing 68 (CCDC68) demonstrates a tumor-suppressive role in pancreatic ductal adenocarcinoma. Oncogene (2015) 34(32):4238–47. doi: 10.1038/onc.2014.357 PMC5153324 25381825

[28] Bai L Yang ZX Liu JS Wang DS Yu HC. Prognostic significance of CCDC137 expression and its association with immune infiltration in hepatocellular carcinoma. Dis Markers (2022) 2022:5638675. doi: 10.1155/2022/5638675 36061359 PMC9433253

[29] Condon KJ Orozco JM Adelmann CH Spinelli JB van der Helm PW Roberts JM. Genome-wide CRISPR screens reveal multitiered mechanisms through which mTORC1 senses mitochondrial dysfunction. Proc Natl Acad Sci USA. (2021) 118(4). doi: 10.1073/pnas.2022120118 PMC7848693 33483422

[30] Wang Z Wang X Zhang N Zhang H Dai Z Zhang M. Pentraxin 3 promotes glioblastoma progression by negative regulating cells autophagy. Front Cell Dev Biol (2020) 8:795. doi: 10.3389/fcell.2020.00795 32984316 PMC7479068

[31] Chen Y Pei M Li J Wang Z Liu S Xiang L. Disruption of the CCDC43-FHL1 interaction triggers apoptosis in gastric cancer cells. Exp Cell Res (2022) 415(1):113107. doi: 10.1016/j.yexcr.2022.113107 35306026

[32] Russell JO Camargo FD. Hippo signalling in the liver: role in development, regeneration and disease. Nat Rev Gastroenterol Hepatol (2022) 19(5):297–312. doi: 10.1038/s41575-021-00571-w 35064256 PMC9199961

[33] Fu Z Fan X Wang X Gao X. Cistanches Herba: An overview of its chemistry, pharmacology, and pharmacokinetics property. J Ethnopharmacol (2018) 219:233–47. doi: 10.1016/j.jep.2017.10.015 29054705

[34] Ma X Liu J Yang L Zhang B Dong Y Zhao Q. Cynomorium songaricum prevents bone resorption in ovariectomized rats through RANKL/RANK/TRAF6 mediated suppression of PI3K/AKT and NF-kappaB pathways. Life Sci (2018) 209:140–8. doi: 10.1016/j.lfs.2018.08.008 30092296

[35] Gao X Liu Y An Z Ni J. Active components and pharmacological effects of cornus officinalis: Literature review. Front Pharmacol (2021) 12:633447. doi: 10.3389/fphar.2021.633447 33912050 PMC8072387

