# Supplementary figures and images for: CCDC43 as a potential therapeutic target of Tian Yang Wan for the treatment of hepatocellular carcinoma by activating the hippo pathway
Source: Front Oncol. 2023 Aug 8;13:1232190. doi: 10.3389/fonc.2023.1232190 (PMC10444197; doi:10.3389/fonc.2023.1232190)

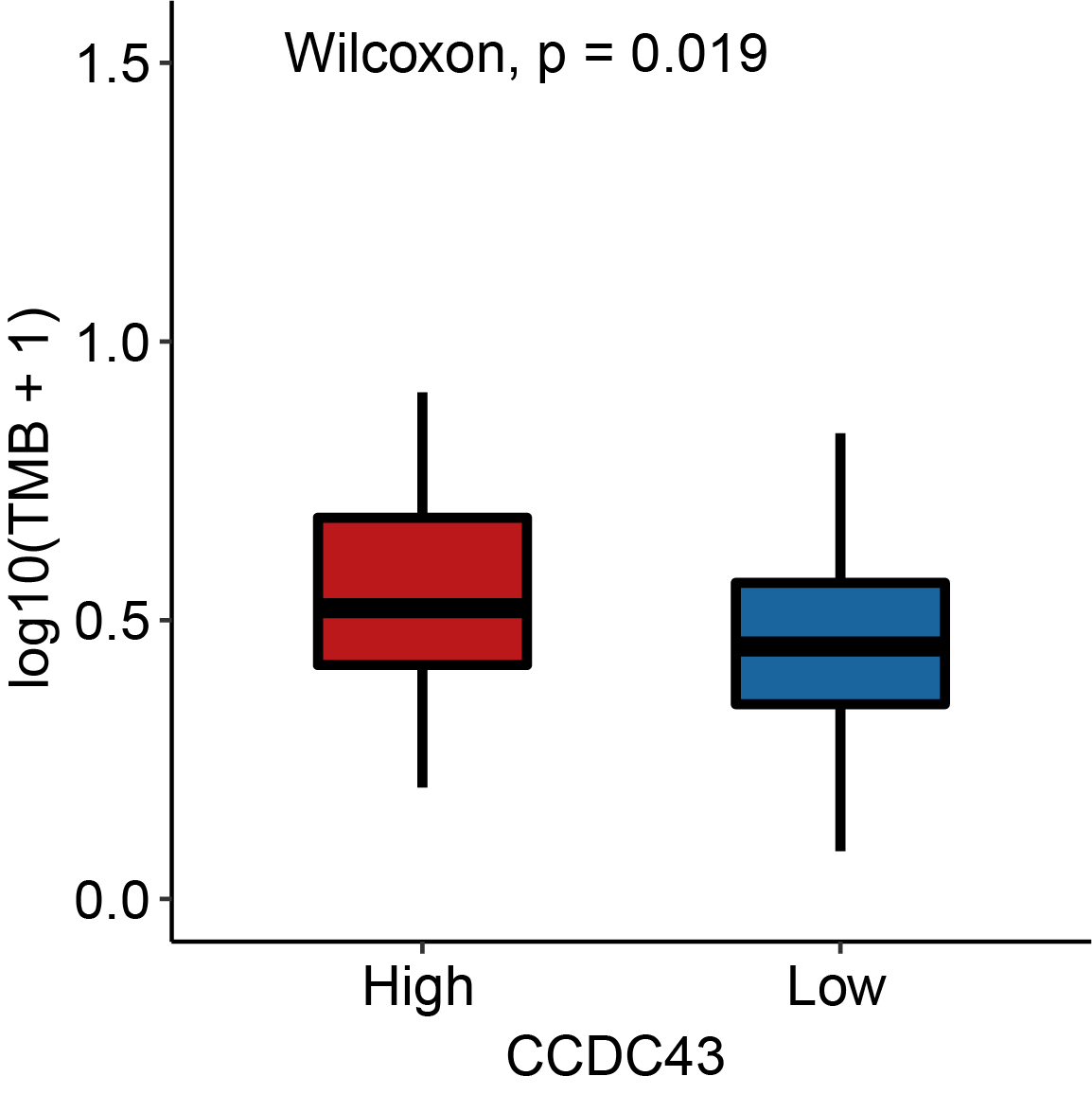

Supplement: Supplementary Figure 1 — Box plot of TMB levels between high and low CCDC43 expression groups. [file Image_1.jpeg]

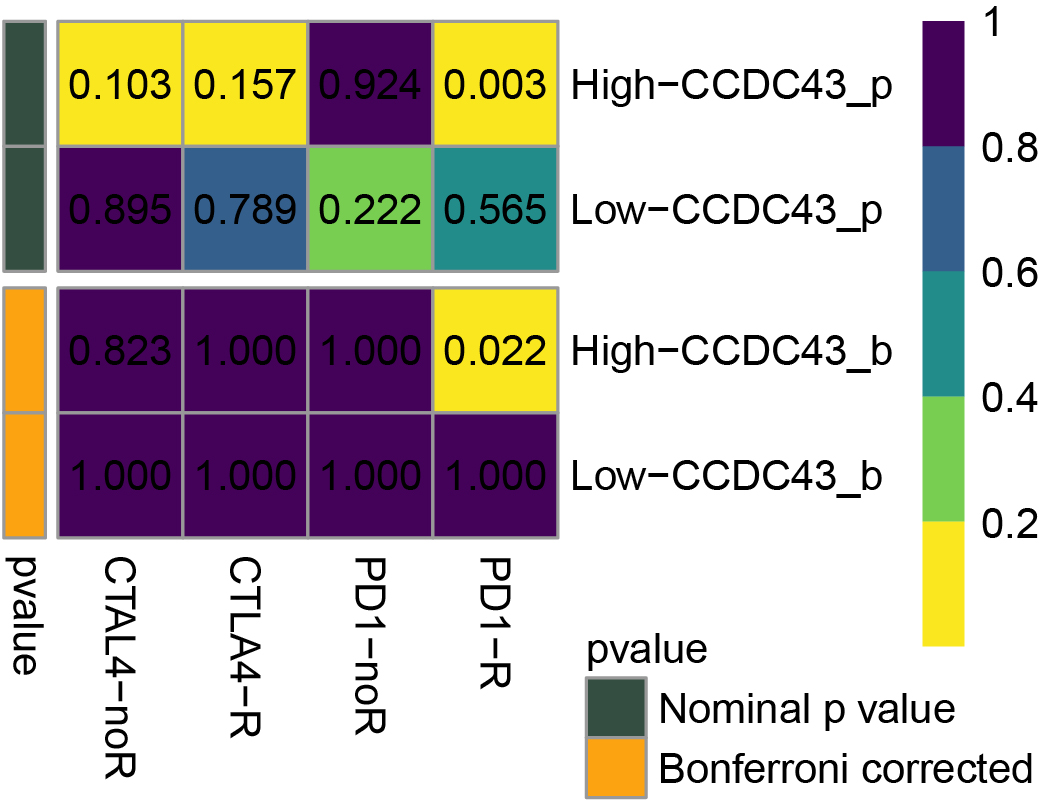

Supplement: Supplementary Figure 2 — SubMap analysis of CCDC43 expression levels in HCC. [file Image_2.jpeg]
